# Mobile phone imaging and cloud-based analysis for standardized malaria detection and reporting

**DOI:** 10.1038/srep28645

**Published:** 2016-06-27

**Authors:** Thomas F. Scherr, Sparsh Gupta, David W. Wright, Frederick R. Haselton

**Affiliations:** 1 Department of Biomedical Engineering, Vanderbilt University, Nashville, TN; 2 Department of Chemistry, Vanderbilt University, Nashville, TN.

## Abstract

Rapid diagnostic tests (RDTs) have been widely deployed in low-resource settings. These tests are typically read by visual inspection, and accurate record keeping and data aggregation remains a substantial challenge. A successful malaria elimination campaign will require new strategies that maximize the sensitivity of RDTs, reduce user error, and integrate results reporting tools. In this report, an unmodified mobile phone was used to photograph RDTs, which were subsequently uploaded into a globally accessible database, REDCap, and then analyzed three ways: with an automated image processing program, visual inspection, and a commercial lateral flow reader. The mobile phone image processing detected 20.6 malaria parasites/microliter of blood, compared to the commercial lateral flow reader which detected 64.4 parasites/microliter. Experienced observers visually identified positive malaria cases at 12.5 parasites/microliter, but encountered reporting errors and false negatives. Visual interpretation by inexperienced users resulted in only an 80.2% true negative rate, with substantial disagreement in the lower parasitemia range. We have demonstrated that combining a globally accessible database, such as REDCap, with mobile phone based imaging of RDTs provides objective, secure, automated, data collection and result reporting. This simple combination of existing technologies would appear to be an attractive tool for malaria elimination campaigns.

Recent efforts to control malaria have been remarkably successful in lowering the burden that disease-endemic countries face, as evidenced by a 30% decrease in case incidence since 2000^1^. Amongst other measures, these efforts have included the deployment of millions of immunochromatographic rapid diagnostic tests (RDTs)^1^. These tests are easy-to-use, inexpensive, and have a simple workflow that can be completed by minimally trained healthcare workers^2^,^3^,^4^,^5^. This makes RDTs a particularly useful tool for identifying malaria in patients presenting with symptoms common to several tropical diseases.

Significant progress has been made in reducing the incidence of malaria, yet malaria led to 584,000 deaths in 2013 and 3.3 billion people remain at risk of infection^1^. So while RDTs have had a positive impact, they are still hindered by the limited training that health workers administering these tests recieve^6^, and elimination campaigns will require improvements to reduce errors in visual interpretation of RDTs. Alternative but more quantitative and sensitive techniques for the detection of malaria are labor intensive, expensive and require trained workers. For example, microscopic inspection of a blood smear remains the gold standard of malaria diagnosis^7^,^8^, but it requires a microscope and trained personnel^9^. Nucleic acid amplification by polymerase chain reaction has sensitivity down to 1 parasite per microliter of blood^10^,^11^,^12^,^13^, but it has significant infrastructure requirements and is subject to reagent and power availability. While both of these methods have lower detection limits relative to RDTs, they lack mobility and ease-of-implementation that renders them impractical at the point-of-care. To overcome these challenges, RDT “readers” have been developed to provide a means to objectively interpret and quantitatively measure RDT results^14^,^15^, however, readers are costly and typically lack mobility for elimination campaigns – two of the key attributes of RDTs. Improvements to RDT readers that maintain or improve RDT sensitivity, lower cost, and include results reporting, would make RDT readers an indispensable asset in malaria elimination campaigns.

Though accurate diagnosis of malaria is improved with more sensitive tests, test result reporting is still in need of a solution that works within the different levels of medical record keeping. In a report on a malaria sentinel surveillance case study, Yukich *et al*. provide an excellent description of the challenges in this information workflow^16^. At the most local scale, paper records and registries are considered the gold standard; aggregated data is compiled weekly or monthly and passed along the reporting chain hierarchy; at each level the data is further aggregated, until it reaches the most central level—typically a national Ministry of Health. These results allow for data-driven decisions on the allocation of resources^17^, but poor data recording and supervision at the local level results in inadequate and incomplete results reporting. On a much larger scale, inconsistent reporting is one of the reasons that the World Health Organization struggles to accurately assess malaria trends in 15 countries in West Africa, 9 countries in Central Africa, and 7 countries in East and Southern Africa^1^. As the importance of this challenge has come to light, surveillance programs have improved in recent years and are beginning to accept more modern technologies including SMS-based results reporting from rural health facilities^16^,^17^,^18^,^19^. As this gains more widespread acceptance, RDT readers that immediately report their results can reduce errors and provide near real-time results for critical healthcare resource deployment decisions.

Mobile phones are one means to achieve the test interpretation and reporting goals. They provide the test interface required for analysis and also have demonstrated reporting capabilities, for example: monitoring travel patterns for epidemiological studies^20^,^21^ and for improved case detection and management^22^. Laboratory-based tests that utilize a mobile phone camera have been developed to image colorimetric assays in a 96-well plate^23^, and to connect mobile phones to conventional microscopes and transmit images of blood films^24^. In an effort to move away from the requirement of an equipped laboratory, optical systems that transform a mobile phone camera into a stand-alone microscope for the diagnosis of global health diseases have been developed^25^,^26^. Other adapters and clip-on fittings have been used to image rapid diagnostic tests^27^,^28^,^29^, but these have never been critically evaluated to justify the necessity for specialized accessories.

A potential solution to the challenge of integrated results reporting, REDCap (www.projectredcap.org) is a freely available secure web application designed to manage online surveys and databases. The REDCap consortium includes 1,678 active institutional partners across 96 countries, including many in low resource countries, and it has an ideal infrastructure for maintaining patient test results^30^. The inclusion of REDCap allows for global deployment, secure collection and transmission of patient demographic information, as well as test result information, all of which will be critically important as disease control strategies shift to elimination campaigns.

In this work, we demonstrate that the camera within a mobile phone provides an objective recording of an RDT, and the web access on the phone enables linking to REDCap for accurate and immediate results reporting. We compare the limits of malarial parasitemia detection using a standard mobile phone camera, a commercial lateral flow reader, and visual inspection. Images are collected with a mobile phone and uploaded to the open access and widely deployed REDCap database, and then downloaded to a server for image analysis. The use of an established, open-source, online database manager makes this analysis readily available for further field usage, such as studies involving disease epidemiology and the tracking of malaria infections for elimination campaigns.

## Materials and Methods

### Ethics statement

Pooled human whole blood was purchased from Bioreclamation IVT for use in this study. Blood collection followed protocols and guidelines that were approved by Schulman Associates IRB, Inc. (IRB number 201209942).

### Materials

Paracheck Pf rapid diagnostic tests (Cat# 30301025) were acquired from Orchid Biomedical Systems (Goa, India). D6 *Plasmodium falciparum* stock culture was prepared in the Wright laboratory at a stock concentration of 18,450 parasites/μL. Dilutions of the stock culture were made into pooled human whole blood purchased from BioreclamationIVT (Cat# HMWBCPD, BioreclamationIVT, Baltimore, MD), resulting in parasite concentrations of 0, 12.5, 25, 50, 100, 250, and 500 parsites/μL. Each concentration was tested in triplicate, resulting in 21 total RDTs. The RDTs were quantitatively analyzed with an ESEQuant Lateral Flow Reader (LFR) (Qiagen, Stoackach, Germany) and an iPhone 5s (Apple, Cupertino, CA).

### Rapid diagnostic testing procedure

The RDT manufacturer’s recommended operating procedure was used, with the following modifications to make a direct comparison of the same RDTs between the three quantification methods at an equivalent time point. Briefly, 5 μL of parasite-spiked blood was added to the conjugate pad of the test and 3 drops of running buffer (unmodified from that supplied with the RDTs) was added to the buffer pad. While the manufacturer recommends analyzing the test after 30 minutes, we allowed the tests to completely dry before analysis. After the test dried, approximately 3 hours, the immunochemical reaction on the strip has run to completion and is no longer changing. As the RDT develops, there is an initial time period where the test line intensity changes substantially. After this initial development, changes to the RDT signal intensity are much slower. We allowed the extra time for drying in these experiments only to facilitate the analysis of multiple RDT evaluation methods and do not envision it as a necessary step in the future.

### Visual test interpretation

After the RDTs dried to completion, the tests were fixed to individual 3”×5” white notecards and randomly ordered. Laboratory researchers (n = 19) in the Department of Biomedical Engineering and the Department of Chemistry at Vanderbilt University were asked to identify positive or negative test results. The researchers were split into two groups: those with greater than 30 minutes of experience with rapid diagnostic tests, and those with less than 30 minutes of experience with rapid diagnostic tests. The researchers were provided with basic instruction on interpretation of the RDTs from the foil package of the rapid diagnostic test. Researchers were instructed to spend no more than 30 seconds interpreting any individual test, and to evaluate each test independently of previous tests. Reviewers were blinded as to the parasite concentration in the blood that was added to the test, and the only identifying mark on the notecard was a single letter that was used for recording purposes. They were provided with a sheet of paper and asked to mark each test as positive or negative. For analysis, positive scores were given a value of 1, and negative scores given a value of 0. Combining the results of each individual test, the average and standard deviation across each cohort were calculated at each concentration.

### Lateral flow reader and image collection

**Figure 1:**
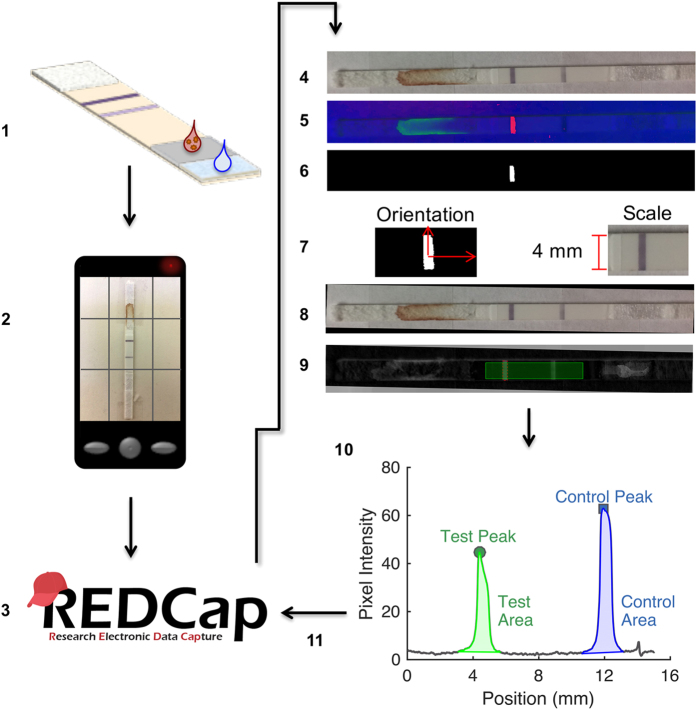
The diagnostic strategy workflow and image-processing algorithm. The REDCap logo is reproduced with permission from REDCap.

RDTs were analyzed using the ESEQuant LFR with the following settings: number of scans set to 1, signal noise set to 30 mV, reflective reading on the E1/D2 channel, and scan length of 60 mm. The peak area was integrated using a fixed baseline, and including 1.5 mm upstream and downstream of the peak in the line scan. The same RDTs were imaged using an iPhone 5s under ambient laboratory lighting conditions. The test strip was removed from its plastic housing, and fixed to a sheet of paper with double-sided tape. This was only done to facilitate the multiple reading methods and is not necessary for our image processing. The RDTs were only coarsely oriented with the control line to the left of the test line. To minimize shadowing, the paper was held vertically during imaging and the phone was manually held 3.5 inches from the paper. There was some slight variation in imaging distance, and its impact is addressed in the Discussion section. There were three RDTs for each concentration, and they were captured in a single image. The individual RDTs were cropped, and the resulting images were subsequently uploaded to REDCap along with demographic patient information for further analysis (see Supplementary Fig. S1). For demonstration purposes, the patient information that was collected was: name, gender, age, address, test location, and test brand. The end-user workflow, including the administering of the test, is summarized in steps 1–3 of Fig. 1.

### Image processing in MATLAB

A schematic of the automated image processing software is shown in steps 5–11 of Fig. 1. Images were downloaded from REDCap to a local computer for automated analysis with MATLAB’s (v8.5 MathWorks, Natick, MA) Image Processing Toolbox (v9.2). While the Image Processing Toolbox makes MATLAB an ideal software environment for the development of advanced image processing algorithms, the algorithms could readily be ported to any freely available and widely-used programming language (Java, C++, Python). The steps in the image processing algorithm are detailed as follows. Since the RDTs were only coarsely aligned, and the pictures were taken from slightly different distances, a measure of RDT orientation and scale were needed. The color and position of the control line was relatively consistent; this made it an easy feature to identify. The raw RGB (red-green-blue) image was converted to a HSV (hue-saturation-value) colormap. The purple color of the control line was identified and a binary threshold that isolated this component of the RDT was created. From this, the incident angle that the RDT makes with the horizontal axis was calculated, as well as the scale of the image (we assumed that the width of the control line was the full width of the RDT membrane). The raw image of the RDT was rotated to orient the control line normal to the x-axis of a Cartesian plane. The background of the image was subtracted using a tophat morphological operation with a disc-shaped structuring element of 2 mm in size. A perpendicular line scan, of a given width, was drawn that spans 3 mm downstream of the control line and 12 mm upstream of the control line. The line scan averaged the gray scale pixel intensity of the background-subtracted image over the width of the scan, as a function of position. The greatest pixel intensity came from the peak of the control line, and made the control line easy to find along the line scan. Pixel intensity was integrated from 1.25 mm downstream of the control line peak to 1.25 mm upstream of the control line peak. A linear relationship between these two points was used to subtract a baseline from the peak. With Paracheck RDTs, the test line is approximately 8 mm upstream of the control line. A search was performed over a 2.5 mm distance centered 8 mm upstream of the control line, to look for the largest pixel intensity in that window on the line scan. Once this test line peak was identified, it was integrated from 1.25 mm downstream of the test line peak to 1.25 mm upstream of the test line peak. The integrated peak areas were then uploaded to REDCap and associated with the raw image and patient demographic information.

### Statistical analysis

A visual limit of detection was determined to be the lowest parasite concentration that resulted in a positive test interpretation by at least 90% of the evaluators. For the lateral flow reader and the image processing test interpretation, the limit of detection was calculated using the method established by Armbruster and Pry^31^. Briefly: a limit of blank was calculated, which is the apparent integrated intensity of a parasite-free blood sample. This limit of blank was used to calculate the limit of detection, which is the lowest parasite concentration that can reliably be distinguished from the limit of blank. A linear regression was used to relate integrated intensity values to parasite concentrations. To determine the signal-to-noise ratio, the “signal” was the integrated pixel intensity of the test line peak of the RDT. The “noise” was the mean value of the integrated test peaks of the 0 parasite per μL RDTs.

### Simulated low resolution images

In order to determine the impact of camera resolution on the effectiveness of the automated image analysis program, low resolution images were simulated of high (500 parasites per microliter) and low (25 parasites per microliter) parasite concentration RDTs. The simulation was accomplished by first resizing the existing 8 megapixel images by the appropriate factor (e.g. in order to create a 4 MP image, a factor of ½ was used). Then, the smaller image was resized back up to the pixel size of the original 8 MP image (e.g. the smaller 4 MP image was scaled up by a factor of 2). This effectively allowed for the loss of information equivalent to a lower resolution image. The scaling was done equally in both dimensions. This was repeated for a series of resolutions: 8 MP (original, no modification), 4 MP, 2 MP, 1 MP, 0.5 MP, 0.3 MP. These images were then analyzed using the automated image analysis program. While subsampling high-resolution images does not take into account sensor blurring or random sensor noise^32^, it is an appropriate approximation of images collected with a low resolution camera.

## Results

### Visual test interpretation

**Figure 2:**
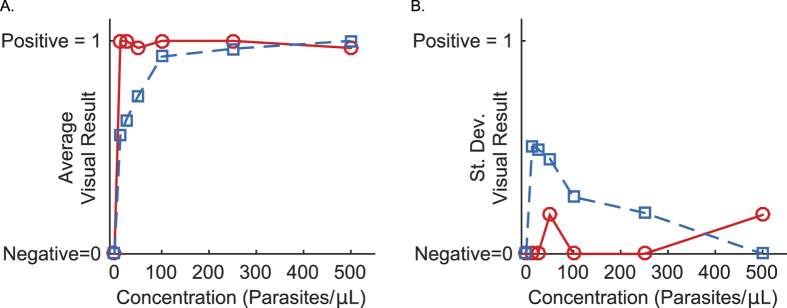
(**A**) The average visual interpretation scores, and (**B**) the standard deviation of the visual interpretation scores of experienced observers (red circles, solid line, n = 10) and novice observers (blue squares, dashed line, n = 9).

The importance of previous experience in lateral flow assay interpretation, especially at low concentrations, is shown in Fig. 2. With novice interpreters, the positive tests (spiked with a known concentration of parasite culture) with parasite concentrations of less than 100 parasites per microliter had average scores less than 0.75. The disagreement between the “inexperienced” observers is at its largest at 12.5 parasites per microliter, where the standard deviation amongst that cohort was 0.192. The limit of detection for the inexperienced readers, where there was greater than 90% agreement on a positive test, was 100 parasites per microliter. On the other hand, the cohort with 30 minutes of prior experience was nearly perfect in their correct identification of positive tests. The visual limit of detection for the experienced group was 12.5 parasites per microliter, the lowest parasite concentration that was evaluated. Both cohorts were unanimous in the identification of the three negative tests. These results are summarized in Table 1: the sensitivity of visual test interpretation increased from 80.2% to 98.9% in those that had more than 30 minutes of experience reading RDTs. Regardless of experience level, all users were 100% accurate in identifying true negatives.

### Lateral flow analysis using a commercial reader

**Figure 3:**
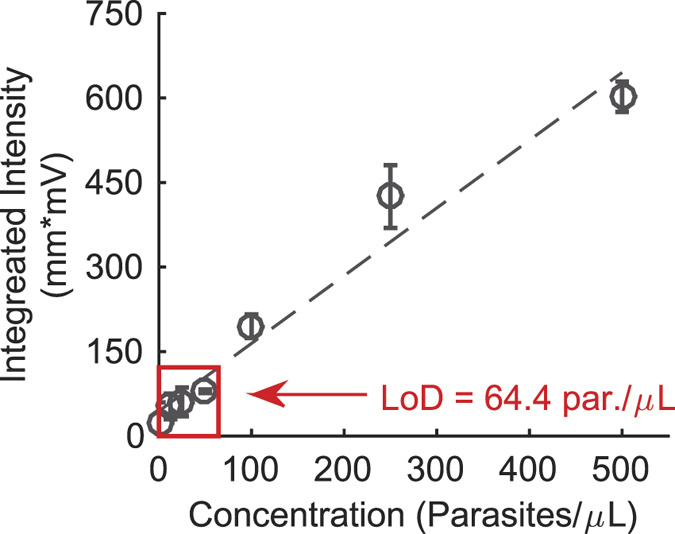
Integrated intensities (mean +/− standard deviation, n = 3 for each concentration) used for the Qiagen lateral flow reader limit of detection determination. The dashed line is the linear fit of the data between 0 parasites per microliter and 500 parasites per microliter.

The serial dilution of rapid diagnostic tests was completed using parasite-spiked blood and analyzed using a commercial lateral flow reader. The purple control lines are visually similar in color and intensity regardless of parasite concentration. The test lines are qualitatively more intense at higher concentrations, but they are more difficult to distinguish at concentrations of less than 50 parasites per microliter. Using the Qiagen LFR software, the peaks of the test lines were analyzed. Figure 3 shows the integrated area under the test line peak as a function of parasite concentration. The limit of detection was calculated to be 64.4 parasites per microliter of blood.

### Limit of detection from mobile phone images

**Figure 4:**
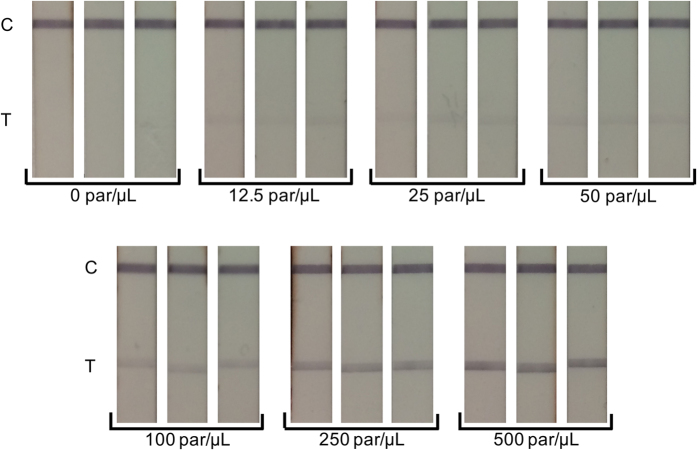
RDT images at concentrations of 0, 12.5, 25, 50, 100, 250, and 500 parasites per microliter of blood. The control lines and test lines of the RDTs are identified by C and T, respectively. These images have been manually cropped, re-sized, and aligned for uniformity, but not contrast enhanced or brightened.

**Figure 5:**
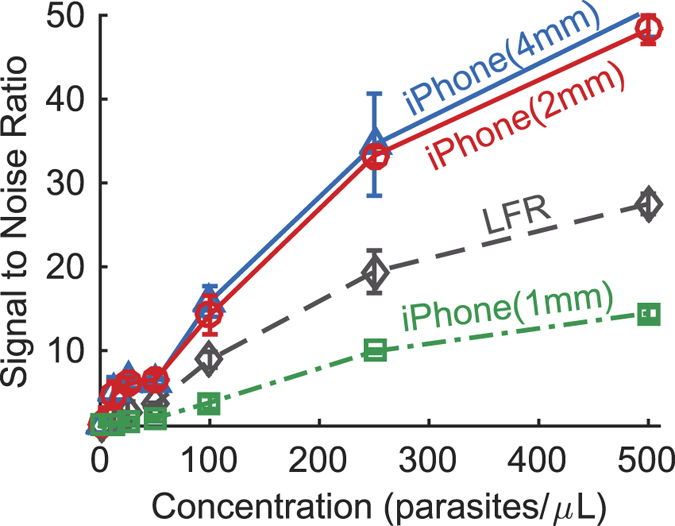
The signal to noise ratio of the integrated test peaks from the lateral flow reader and selected line scan widths (in parentheses) using the mobile phone.

Images of the individual tests taken with a mobile phone are shown in Fig. 4. These images have been manually oriented and scaled for the sake of visualization, but not manipulated in any other way. The raw images (shown in Supplementary Fig. S1) were downloaded from REDCap and processed with the MATLAB image processing program. The limit of detection for the iPhone with line scan widths of 0.5 mm, 1 mm, 2 mm, 3 mm, and 4 mm, are shown as Supplementary Fig. S2, and along with the limit of detection for the commercial lateral flow reader, are summarized in Table 2. As the line scan width increased, the calculated limit of detection decreased. The widest line scan tested was 4 mm, and it resulted in a limit of detection of 20.6 parasites per microliter; this is also illustrated through a comparison of the signal-to-noise ratio of the methods, shown in Fig. 5. The 4 mm line scan from the mobile phone had the highest signal to noise ratio at each parasite concentration evaluated. Although the signal to noise ratios are similar, the limit of detection of the 4 mm line scan is nearly 20 parasites per microliter better than the limit of detection of the 2 mm line scan. The limit of blank, the smallest integrated peak area (with units of intensity*mm) that is statistically detectable, is calculated to be 5.14 for the 1 mm line, compared to 2.63 for the 2 mm line, 1.88 for the 3 mm line, and 1.58 for the 4 mm line; an increased limit of blank results in a poorer limit of detection. While the limit of detection with a 4 mm is the lowest, a portion of the line scan extends beyond the width of the test strip (see Supplementary Fig. S3). This is likely due to the test line and control line not being perfectly orthogonal to the nitrocellulose membrane during the manufacturing process; as such, we would recommend sacrificing some improvement in the limit of detection for robustness in the image processing algorithm by using a line scan smaller than the width of the RDTs.

### Simulated low resolution images

Down sampling an image, followed by scaling it back to its original size has the undesirable effect of loss of pixel information. This was used to simulate the impact of a low resolution camera on our automated image processing algorithm. The resulting low resolution images qualitatively show little information loss after scaling down from 8 MP to 2 MP. Simulated low resolution images for 1 MP, 0.5 MP, and 0.3 MP are shown in Supplementary Figs S4 and S5, along with the corresponding line scans. The automated analysis program was able to generate a result for each low resolution image. Inspection of the low resolution images of the 500 parasites per μL RDT show that the program correctly located the control line and rotated the image correctly to generate a perpendicular line scan. The line scan does not appear to show signs of “pixelation”, despite severe blurring in the 0.3 MP image. This indicates that as long as the quality of the image is sufficient to allow for detection of the control line, the image analysis program can integrate the peaks that result from the line scan.

The 0.3 MP image at 25 parasites per μL show that the program failed to correctly find the control line. The algorithm detected the location of the control line, as seen by the localization of the line scan. However, it was unable to appropriately determine the orientation and size of the control line; this resulted in an incorrect rotation of the image, and a short and narrow scan that did not capture the test line. For quality assurance, the program should be revised to alert the user to a potential error when atypical control line sizes or orientations are detected. It is important to note that this failure was at a low parasite concentration, and a camera resolution of 0.3 MP. A small increase in pixel information to 0.5 MP allows the algorithm to detect even this faint test line.

## Discussion

As efforts shift from malaria control to malaria elimination, diagnostic tools will need modification to provide objective results and interface with new reporting needs. By design, lateral flow assays are inexpensive and easy to use. While this has led to large-scale deployment, it limits their utility in elimination campaigns that require consistent interpretation and high levels of coordination among manufacturers, government health agencies, and field healthcare workers. Improvements in diagnostic strategies are needed that utilize the simplicity of the lateral flow assay, but provide an unambiguous result and integrate features such as automated test result reporting, usage monitoring, and effective healthcare resource allocation.

One of the most critical necessities of any new diagnostic strategy is to provide an objective diagnosis with the same or better sensitivity than current methods. The visual inspection of RDTs is inherently subjective and, even for our small sample size (n = 10 experienced readers, n = 9 inexperienced readers), results show that previous experience and training in test interpretation correlate with a more accurate test reading. Although this result is expected and has been demonstrated on a larger scale^33^, it underlines a clear challenge that malaria elimination campaigns face. This result could become more important in regions with declining prevalence of malaria; as the incidence of positive tests decreases, those reading the tests will have less experience visualizing positive RDTs as demonstrated in mass screening efforts in low transmission settings^34^. Approaches that provide an accurate and objective reading, especially at low levels of parasite in blood, offer a substantial improvement when training of health workers is limited.

There were two false negatives in the experienced reader group, one at the low parasitemia of 50 parasites per μL and, interestingly, the other at 500 parasites per μL. The former was a true misinterpretation of the test line; the reader could not identify a faint test line. The false negative at 500 parasites per μL was a reporting error; the user stated that he correctly identified the test, but unintentionally recorded the wrong diagnosis. This illustrates that even trained healthcare workers can suffer from reporting errors, and technologies that can remove these errors have a clear utility in the field.

Both the mobile phone based reader and the commercial RDT reader used in this study, couple the inexpensive and easy-to-use lateral flow assay with a quantitative and objective test interpretation. However, the commercial reader is expensive (>$3,000), requires manual user input to identify the test line and control line, and has limited built-in reporting capabilities. On the contrary, our mobile phone based approach relies on a relatively inexpensive mobile phone camera, is fully automated, and combined with REDCap demonstrates the potential for automated results analysis and reporting. While the mobile phone image processing system has shown improved detection, it will still inherently rely on the sensitivity of the RDT being used.

The method presented in this report works with an unmodified mobile phone, and relies on software to perform its analysis with “imperfect” images. Other work has employed mobile phones, paired with additional housings or adapters, to image lateral flow strips. However, these additional requirements make them less likely to gain widespread adaption. Across the images collected for this work, there were moderate variations in lighting, imaging distance, and test orientation. Despite these variations, the algorithm generated reproducible results. Furthermore, we have simulated images of RDTs from low resolution cameras and found that our algorithm reliably works for camera resolutions as low as 0.5 MP, at both a high and low parasite concentration. While the iPhone 5s used in this work has a resolution of 8 MP, we expect that any modern mobile phone camera will capture images at sufficiently high resolution. In the future we will quantify the effect of more drastic variation in lighting conditions. We anticipate that with appropriate image processing and normalization, quantitative results can still be obtained under field-relevant lighting. In addition, we intend to adapt the algorithm to accommodate multiplexed tests with multiple analyte test lines. This will only require slight programmatic tuning to account for variations in fabrication, namely, test/control line color and spacing.

While images in this study were manually downloaded and then processed with an automated algorithm, integration with REDCap offers the potential for Data-Engineered Triggers. These event-based triggers make feasible a fully automated-environment, where images can be uploaded from a mobile phone and automatically sent to a secure server for analysis. Results can then be automatically sent back from the secure server to the mobile phone. This process can provide near real-time (<5 minutes) feedback, as well as the possibility for asynchronous data collection and analysis in the event of poor internet connectivity. The combination of de-identified patient demographic data, test administrator information, and automated test result reporting has wide-ranging applications from test manufacturing quality control and usage monitoring to real-time malaria outbreak epidemiology modeling and healthcare resource allocation.

## Conclusion

Rapid diagnostic tests are an attractive tool for disease diagnosis, and their low cost, small footprint, and ease-of-use make them an important component of diagnostic strategies in malaria elimination campaigns. In this work, we have demonstrated the efficacy of mobile-phone imaging of RDTs linked to a clinical database management application, such as REDCap, as a potentially powerful diagnostic strategy. Mobile phones are relatively inexpensive and inherently portable. We have demonstrated that an automated image processing algorithm has an improved limit of detection over a commercially available lateral flow reader, and reduced reporting errors inherent in visual test interpretation. A diagnostic strategy that couples an existing RDT test with optical imaging of test results, quantitative test analysis, and automated reporting, would appear to be an ideal combination for malaria elimination campaigns.

## Additional Information

**How to cite this article**: Scherr, T. F. *et al*. Mobile phone imaging and cloud-based analysis for standardized malaria detection and reporting. *Sci. Rep.* **6**, 28645; doi: 10.1038/srep28645 (2016).

## Supplementary Material

Supplementary Information

## Figures and Tables

**Table 1:** Visual limit of detection, sensitivity, and specificity of visual interpretation of tests.

Reader Experience with RDTs	Limit of Detection (parasites/μL)	Sensitivity (True positive rate)	Specificity (True negative rate)
<30 mins (n = 9)	100	80.2%	100%
>30 mins (n = 10)	12.5	98.9%	100%

**Table 2:** Limit of detections calculated as by Armbruster and Pry^31^.

Qiagen ESEQuant	Limit of Blank (mV*mm)	Limit of Detection (parasites/μL)
	84.4	64.4
**Mobile Phone Line Scan Width (mm)**	Limit of Blank (intensity* mm)	**Limit of Detection (parasites/μL)**
0.5	6.38	96.3
1	5.14	71.2
2	2.63	39.6
3	1.88	24.2
4	1.58	20.6
