# Supplementary material for: Mobile phone imaging and cloud-based analysis for standardized malaria detection and reporting
Source: Sci Rep. 2016 Jun 27;6:28645. doi: 10.1038/srep28645 (PMC4921854; doi:10.1038/srep28645)
Supplement: Supplementary Information [file srep28645-s1.pdf]

Mobile phone imaging and cloud-based analysis for standardized malaria detection and reporting

Thomas F. Scherr[1], Sparsh Gupta[1], David W. Wright[2], Frederick R. Haselton*[1,2]

1 Department of Biomedical Engineering, Vanderbilt University, Nashville, TN

2 Department of Chemistry, Vanderbilt University, Nashville, TN

* Corresponding author: rick.haselton@vanderbilt.edu

Supplementary Material

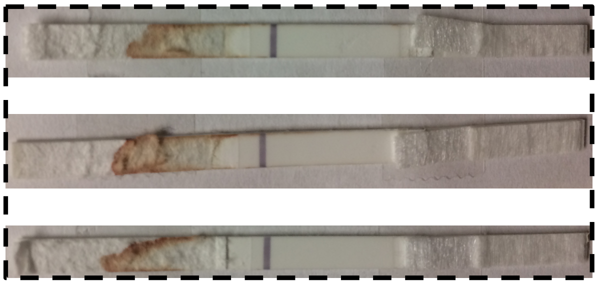

0 par/µL

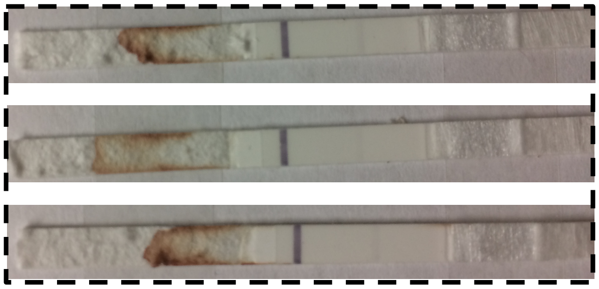

12.5 par/µL

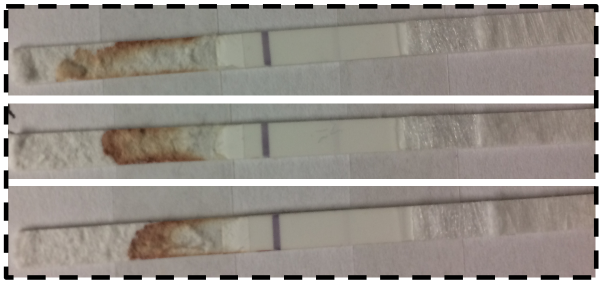

25 par/µL

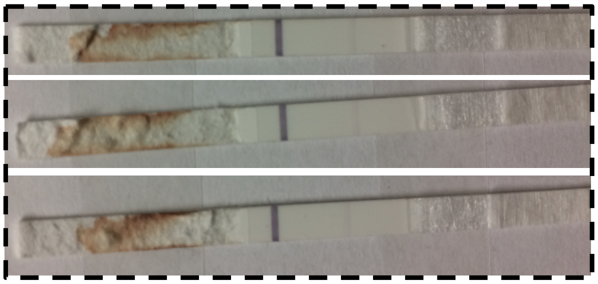

50 par/µL

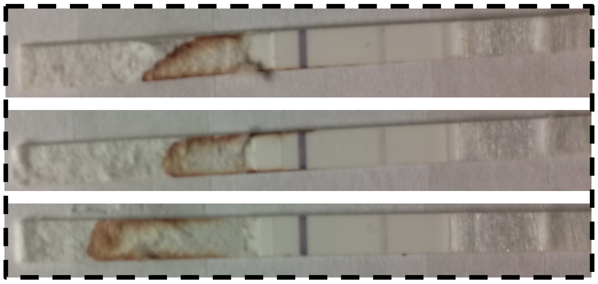

100 par/µL

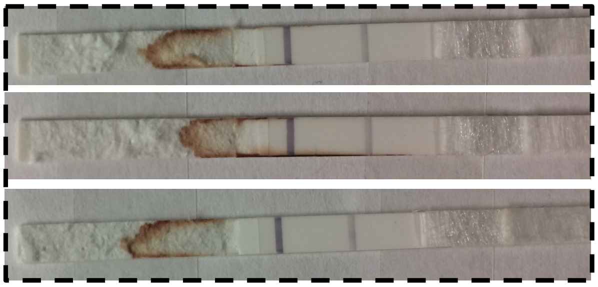

250 par/µL

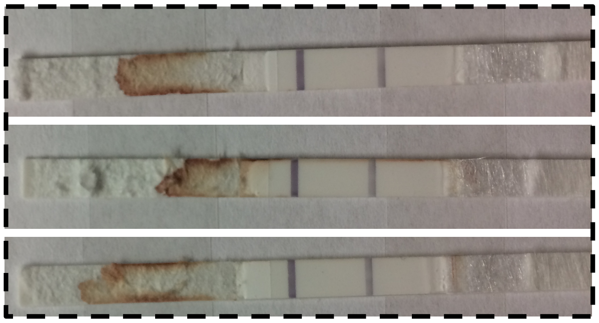

500 par/µL

Supplementary Figure S1. Individual iPhone images of the LFAs used in automated image processing.

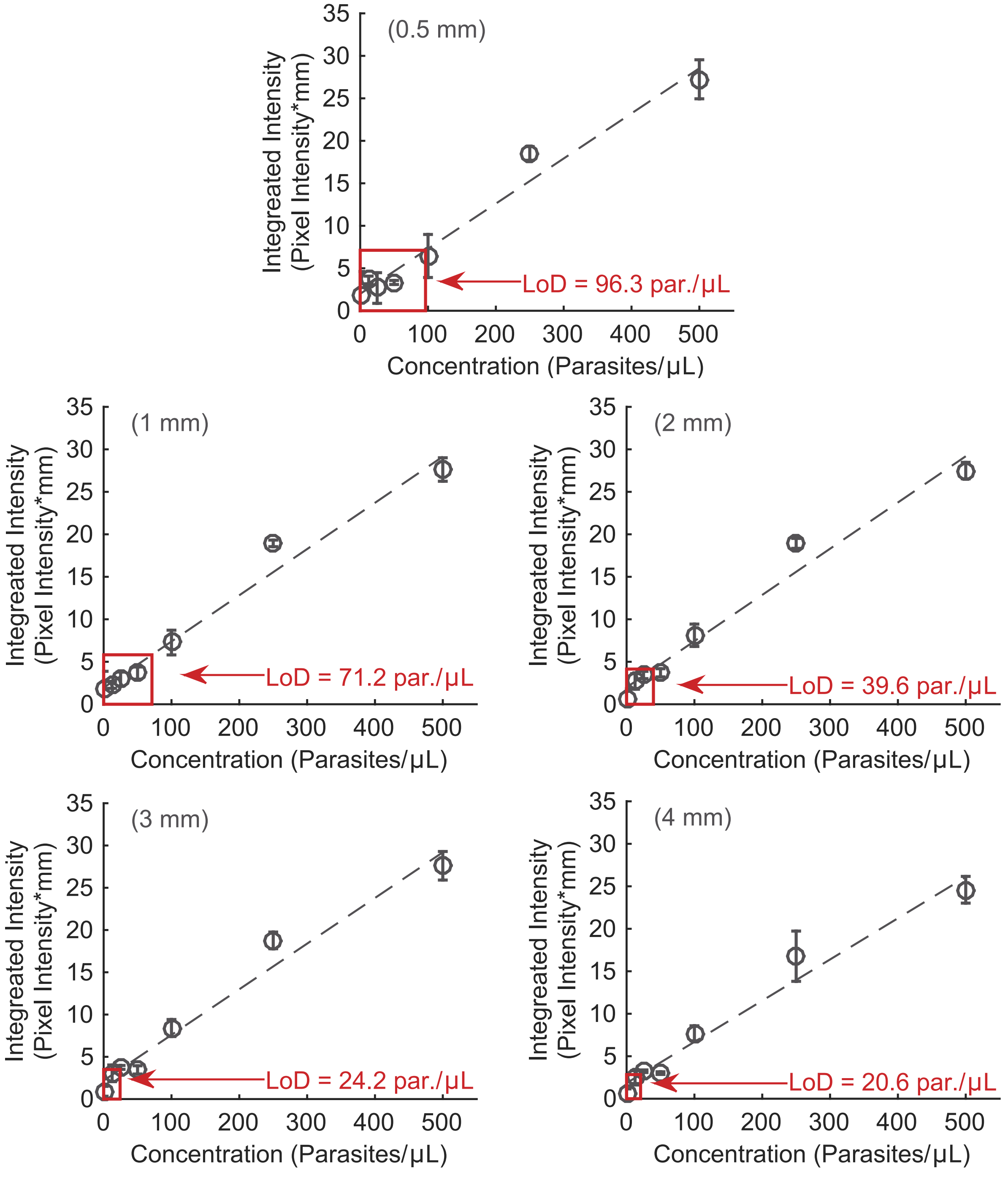

Supplementary Figure S2. Integrated intensities (mean +/- standard deviation) used for the iPhone 5s limit of detection determination for line scan widths of 0.5 mm, 1 mm, 2 mm, 3 mm, and 4mm. The dashed lines are the linear fits of the data between 0 parasites per microliter and 500 parasites per microliter.

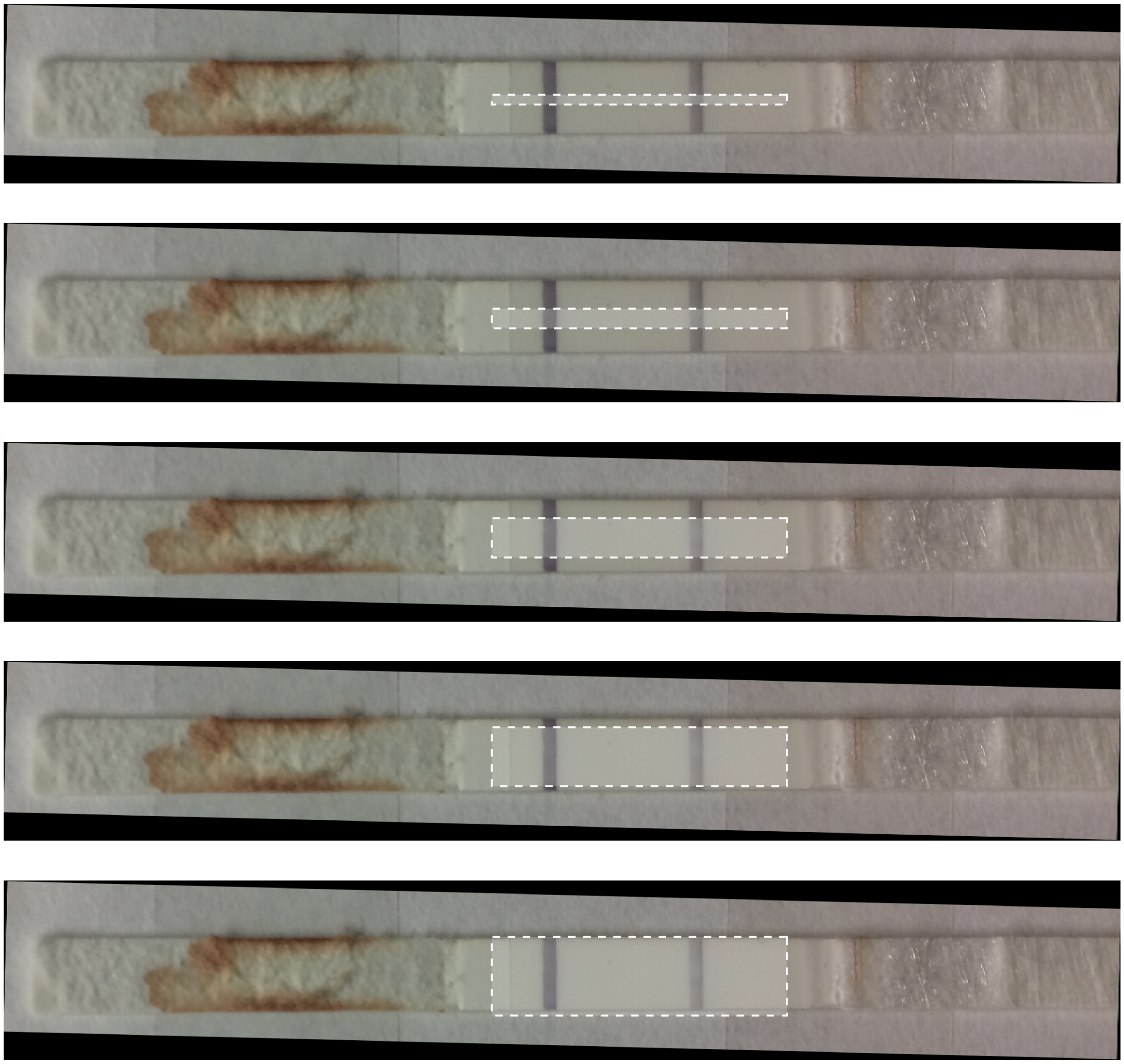

Supplementary Figure S3. The various widths of line scans shown on an image after the control line has been made orthogonal to the image's x-axis. From top to bottom, the line scan widths are: 0.5 mm, 1 mm, 2mm, 3 mm, and 4 mm.

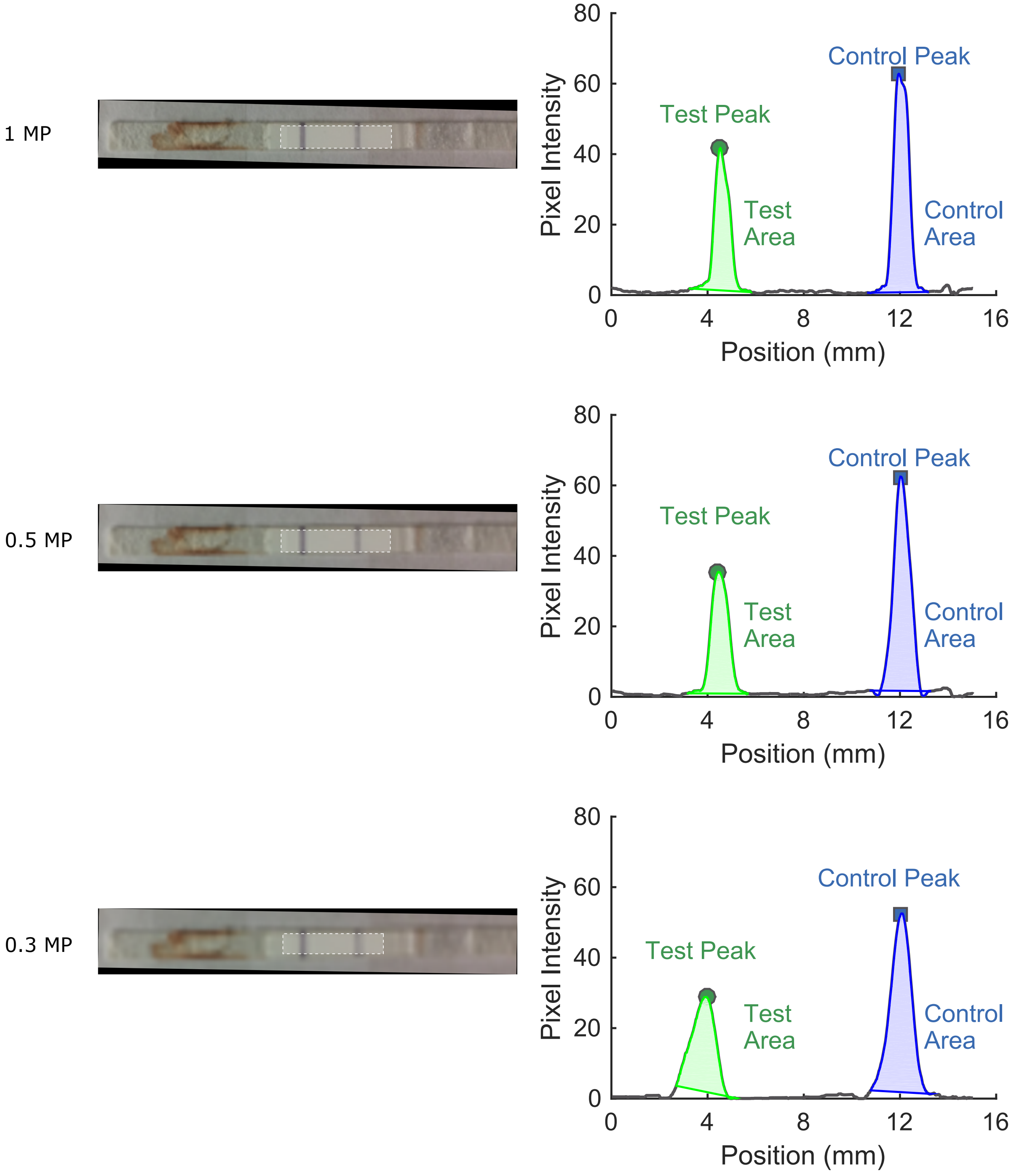

Supplementary Figure S4. Simulated low resolution images of a representative 500 parasite per microliter RDT, after processing with the automated image analysis program. The white box shows the line scan that the image processing program automatically determined. The pixel intensity along each line scan is shown to the right.

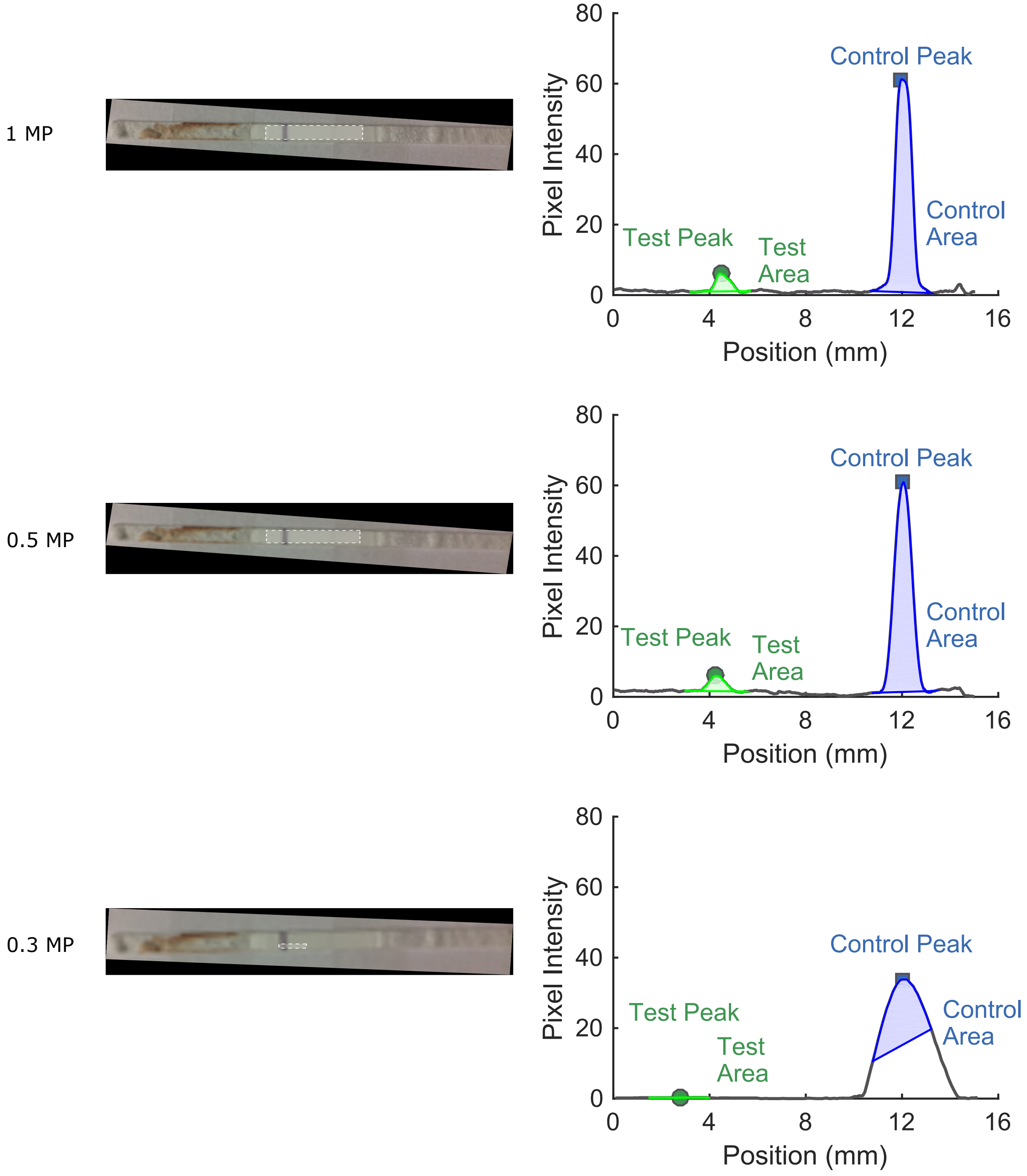

Supplementary Figure S5. Simulated low resolution images of a representative 25 parasite per microliter RDT, after processing with the automated image analysis program. The white box shows the line scan that the image processing program automatically determined. The pixel intensity along each line scan is shown to the right.
